# The Cysteine-Rich Protein Thimet Oligopeptidase as a Model of the Structural Requirements for S-glutathiolation and Oxidative Oligomerization

**DOI:** 10.1371/journal.pone.0039408

**Published:** 2012-06-25

**Authors:** Alberto Malvezzi, Patrícia M. Higa, Antonia T.-do Amaral, Gustavo M. Silva, Fabio C. Gozzo, Emer S. Ferro, Leandro M. Castro, Leandro de Rezende, Gisele Monteiro, Marilene Demasi

**Affiliations:** 1 Instituto de Química, Universidade de São Paulo, São Paulo-SP, Brazil; 2 Instituto Butantan, São Paulo-SP, Brazil; 3 Instituto de Química, Universidade Estadual de Campinas, Campinas-SP, Brazil; 4 Instituto de Ciências Biomédicas, Universidade de São Paulo, São Paulo-SP, Brazil; 5 Faculdade de Ciências Farmacêuticas, Universidade de São Paulo, São Paulo-SP, Brazil; Semmelweis University, Hungary

## Abstract

Thimet oligopeptidase (EP24.15) is a cysteine-rich metallopeptidase containing fifteen Cys residues and no intra-protein disulfide bonds. Previous work on this enzyme revealed that the oxidative oligomerization of EP24.15 is triggered by S-glutathiolation at physiological GSSG levels (10–50 µM) via a mechanism based on thiol-disulfide exchange. In the present work, our aim was to identify EP24.15 Cys residues that are prone to S-glutathiolation and to determine which structural features in the cysteinyl bulk are responsible for the formation of mixed disulfides through the reaction with GSSG and, in this particular case, the Cys residues within EP24.15 that favor either S-glutathiolation or inter-protein thiol-disulfide exchange. These studies were conducted by *in silico* structural analyses and simulations as well as site-specific mutation. S-glutathiolation was determined by mass spectrometric analyses and western blotting with anti-glutathione antibody. The results indicated that the stabilization of a thiolate sulfhydryl and the solvent accessibility of the cysteines are necessary for S-thiolation. The Solvent Access Surface analysis of the Cys residues prone to glutathione modification showed that the S-glutathiolated Cys residues are located inside pockets where the sulfur atom comes into contact with the solvent and that the positively charged amino acids are directed toward these Cys residues. The simulation of a covalent glutathione docking onto the same Cys residues allowed for perfect glutathione posing. A mutation of the Arg residue 263 that forms a saline bridge to the Cys residue 175 significantly decreased the overall S-glutathiolation and oligomerization of EP24.15. The present results show for the first time the structural requirements for protein S-glutathiolation by GSSG and are consistent with our previous hypothesis that EP24.15 oligomerization is dependent on the electron transfer from specific protonated Cys residues of one molecule to previously S-glutathionylated Cys residues of another one.

## Introduction

Thimet oligopeptidase (EC3.4.24.15; EP24.15) is a thiol-rich metallopeptidase ubiquitously distributed in mammalian cells [1], [2]. EP24.15 has been shown to play an important intracellular role in the degradation of peptides released by the 26 S proteasome [3]–[7]. The enzyme is prone to oxidative oligomerization through the formation of interprotein disulfides involving specific Cys residues [8], [9]. It possesses 15 Cys residues and no intra-protein S-S bond. We had already demonstrated that EP24.15 is modified both *in vivo* and *in vitro* by S-glutathiolation and that the formation of intermolecular oxidative crosslinking and subsequent oligomerization is triggered by S-glutathiolation [10]. Moreover, as demonstrated by experiments performed *in vitro*, EP24.15 S-glutathiolation is needed for full peptidase activity. In conclusion, EP24.15 seems to undergo a dynamic mechanism of thiol-disulfide exchange through S-glutathiolation, and the GSSG concentrations necessary to S-glutathiolate EP24.15 and trigger its oligomerization was 10 µM, compatible with GSSG concentrations found in intracellular compartments.

The mixed disulfides of glutathione with proteins (termed protein S-glutathiolation) are observed in many functional conditions, and they accumulate in the mitochondria and cytosol of cells upon oxidative challenge [11]. Protein S-glutathiolation has been proposed as an anti-oxidative defense by protecting either the protein Cys residues from over oxidation or the GSH intracellular pool. Moreover, the S-glutathiolation that occurs during enzyme catalysis and redox signaling has been increasingly accepted as a post-translational protein modification that is dependent on intracellular redox shifts, thereby regulating anti-oxidative cellular responses independent of the global oxidative challenge [11], [12].

There are many mechanisms proposed for protein S-glutathiolation, although most of them were only demonstrated *in vitro* [13]. Mechanisms taking place *in vivo* are still poorly explored. The oxidation of protein Cys residues in sulfenic acid (Cys-SOH) and the subsequent S-glutathiolation of the sulfenic form by the reduced glutathione pool during enzyme catalysis and specific redox signaling have been accepted as commonly occurring events in redox regulation [14]–[19]. On the other hand, protein S-glutathiolation, through the oxidized glutathione species, is thought to be achieved only when the intracellular GSSG pool is increased, which, in turn, occurs upon oxidative stress as the GSSG pool is maintained by cells at low levels under homeostasis [13]. Examples reported in the literature are based on protein S-glutathiolation by the GSSG and are usually related to protein inactivation [11]. In these studies, proteins are usually incubated *in vitro* with high concentrations of GSSG, which would mimic an intense oxidative stressing condition inside cells. Other mechanisms of protein S-glutathiolation have been proposed, such as those resulting from the formation of protein thiyl radicals, followed by the reaction with GSH, and from the S-nitrosoglutathione (GSNO) reaction with the protein sulfhydryl [11], [20]. In fact, the mechanism of S-glutathiolation is dictated by the nature of the protein; in the case of EP24.15, its S-glutathiolation was observed at GSSG concentration as low as 10 µM, compatible with the intracellular milieu in homeostasis conditions [10].

Protein S-glutathiolation depends on the thiol reactivity, pK_a_ and solvent accessibility. The reaction of GSSG with protein thiolate ions (-S^-^) occurs much more readily than with protonated groups (–SH). On the other hand, the formation and stabilization of protein thiolate ions is usually associated with the presence of positively charged groups in the vicinity of the thiol group [21], [22].

Our starting point in the present work was based on previously conducted studies [10]. Those studies revealed that EP24.15 S-glutathiolation by GSSG concentrations as low as 10 µM occurs concomitantly to its oligomerization to the dimer and primarily trimer protein forms. Conversely, using higher concentrations of GSSG (0.5–5 mM), EP24.15 was highly S-glutathiolated and remained in its monomeric form [10]. On the basis of those results, we proposed a mechanism for oligomerization that is dependent on S-glutathiolation at low GSSG concentrations, which would trigger oligomerization through inter-protein thiol/disulfide exchange. Nonetheless, when the number of S-glutathiolated Cys residues was increased (by increasing the GSSG:EP24.15 molar ratio), the protein lost its ability for oligomerization. Notably, when EP24.15 was incubated with H_2_O_2_ and then treated with GSH, it was not S-glutathiolated. Instead, intraprotein disulfide bonds were observed. Thus, the EP24.15 Cys residues that are prone to S-glutathiolation are very reactive toward GSSG.

In the present work, EP24.15 was used as a model to conduct studies searching for protein structural features that might trigger Cys thiolation by GSSG. To accomplish this goal, our approach was first to identify by mass spectrometry EP24-15 S-glutathiolated Cys residues after the incubation of TCEP-reduced protein at a GSSG concentration that concomitantly induces glutathiolation and oligomerization (*e.g.*, 50 µM) and at 1 mM, a concentration at which glutathiolation is increased and oligomerization is inhibited. Afterward, *in silico* GRID and covalent GSH docking to the protein Cys residues were utilized to analyze the solvent accessibility of the Cys residues and to predict the glutathione docking. Finally, a site-specific mutation was utilized to validate some of our findings.

## Materials and Methods

### Reagents

DTT (dithiothreitol), GSSG (oxidized glutathione), and TCEP [Tris (2-carboxy-ethyl) phosphine hydrochloride] were purchased from SIGMA. Anti-GSH antibody was obtained from Virogen and Arbor Assays. Anti-EP24.15 antibody was obtained from Proteimax. Bradford protein assay reagent was purchased from Bio-Rad. All other reagents were of analytical grade.

### Site-directed Mutagenesis

Site-directed mutagenesis of testis rat EP24.15 was performed in pGEX-4t2 (Amersham-Pharmacia Biotech Inc.) using the protocols described by the manufacturer of the Quick-change Site-directed mutagenesis kit (Stratagene, Inc). Oligonucleotide primers 5′ GAG CTA GTG TCC CTG GAG GCG CAG AAG TCC AAC 3′ (sense) and 5′ GTT GGA CTT CTG CGC CTC CAG GGA CAC TAG CTC 3′ (antisense) were used to introduce the R263E point mutation on EP24.15. The plasmid DNA was purified (Mini-Prep, Promega Corp.) and mutations were screened by automatic DNA sequencing, using a MegaBace machine (GE Healthcare). Plasmid DNA containing the desired mutation was purified (Mini-Prep, Promega Corp.), transformed into electrocompetent *Escherichia coli* BL21 cells and plated overnight on plates containing ampicillin to yield single colonies.

### Expression and Purification of Recombinant EP24.15: Wild Type and R263E Mutant

Recombinant proteins (wild type and mutant) were expressed in *E. coli* (XL1-blue or BL21; Stratagene) as glutathione-*S*-transferase (GST) fusion proteins using the expression vector pGEX-4T2 (Amersham Biosciences). Protein purification was initially conducted by affinity chromatography using a glutathione-Sepharose column (Amersham Biosciences) with the respective proteins released from the GST-fusion by cleavage with thrombin (100 U; Amersham Biosciences). Further purification was performed by concentrating the samples in membranes (Millipore, Bedford, MA, USA) with nominal molecular exclusion limits of 50 kDa. The purity of the proteins was analyzed by Coomassie brilliant blue staining after 8% SDS-PAGE. After confirming homogeneity larger than 95% (data not shown), aliquots of proteins were stored at −80°C.

### MS Analyses

After purification and GSSG treatment, the proteins (wild type or the mutant) were subjected to SDS-PAGE. The band of interest was excised from the gel, and after destaining the proteins, EP24.15 was digested with Trypsin Gold (Promega), according to the manufacturer’s protocol. The digested products were recovered and then desalted with C-18 ZipTip resin (Millipore). Another approach was to treat the proteins with GSSG and then perform tryptic digestion. The tryptic fragments were analyzed by LC-MS/MS following separation by reverse-phase HPLC. The additional mass of one glutathione molecule to the cysteine-containing fragments (+305.1 Da) was determined by using the Thermo Finnigan LTQ ion trap mass spectrometer at Instituto de Química – Proteomics Core Facility, UNICAMP, Campinas-SP, Brazil.

### Immunoassays

Immunoblots were performed as described by the protocol enclosed in the ECL™ Western Blotting Systems (GE Biosciences). Membranes were incubated with horseradish-peroxidase-conjugated secondary antibodies, and protein signals were detected using enhanced chemiluminescence Western Blotting Detection Reagents (Amersham Biosciences). The dilutions of the antibodies were as follows: 1∶2000 (anti-EP24.15) and 1∶1000 (anti-GSH). Loading controls were evaluated by anti-EP24.15 when S-glutathiolation was determined as well as by Ponceau S staining.

### Reduction and S-glutathiolation of EP24.15

Preparations of purified recombinant EP24.15 (500–900 µg), the wild type or the mutant protein were incubated with 10 mM TCEP, a specific sulfhydryl reductant. After incubation, protein preparations were filtered through Microcon YM-50 microfilters (Millipore) to completely remove the remaining TCEP by 3–4 cycles of filtration and redilution. Aliquots of these preparations were incubated in the presence of GSSG at indicated concentrations. After measuring the protein concentration, aliquots of reduced and S-glutathionylated protein preparations were used for MS or immunoblot analyses.

### Determination of Protein Concentration

Protein concentrations were calculated by taking the theoretical ε_280_ of EP24.15 (78,240/M deduced by the ExPASy ProtParam tool (http://www.expasy.ch/tools/protparam.html). Bradford reagent was utilized for protein determination as well, using bovine serum albumin (BSA) as a protein standard.

### GRID Methodology

GRID is a fully automated computational method [23], [24]. The 3D structure of the human thimet oligopeptidase 24.15 was used for the GRID analyses, since the rat EP24.15 3D structure is not available. The identity between both sequences, human and rat, is 89%, and nearly all of the cysteine residues are conserved (Fig. S1). The human EP24.15 structure was obtained from the PDB file 1S4B (resolution 2.0 Å), and this structure was considered to be the structure for the wild type protein with no further modifications. Crystal water molecules and ions were removed. The structure of R263E mutant was prepared in the Biopolymer module of the Sybyl 8.0 (Tripos) program (SYBYL, Version 7.0. Saint Louis - USA: Tripos Inc; Copyright 1991–2004, 2004). The side chains of mutant residues were placed in their lowest energy position and were allowed to minimize their energies using the Tripos force field, with Pullman charges and conjugate gradient minimization, keeping all other protein residues rigid.

Wild type and mutant proteins were placed on GRID boxes, and the molecular interaction fields were calculated for each protein using the SH probe (neutral thiol group), as already described [23],[24]. A cubic (10 Å^3^) molecular grid was centered on Cys175, with a grid spacing of 0.11 Å. Minimum energy positions of the SH probe around the Cys175 residue of the wild type and R263E mutant were examined and compared.

### Molecular Dynamics

The 3D structure of human thimet oligopeptidase EP24.15 is available as PDB file 1S4B (resolution 2.0 Å). This structure lacks both N- and C-terminal tails. The C-terminal sequence consists of 10 residues (678-QVEGCEPPAC) and is present in the rat thimet oligopeptidase EP24.15. Because this terminal tail sequence contains two important cysteine residues, a model of this sequence was added to the structure of the human thimet oligopeptidase EP24.15. The rat sequence composed of residues QVEGCEPPAC was added to the Leu677 terminal residue of the human EP24.15. This addition was performed using the Biopolymer module of the Sybyl 8.0 (Tripos) program (SYBYL, Version 7.0., Tripos Inc; Copyright 1991–2004, 2004). Minimization of the added tail sequence was carried out by the Tripos force field using another module of the Sybyl 8.0 program. A molecular dynamics script was then used to simulate the positioning of this tail sequence on the top of human EP24.15. Molecular Dynamics (MD) simulations were performed using the GROMACS5 3.3.3 package with the standard GROMOS96 force field G43a15. Simulations were performed at constant temperature and pressure in a box filled with SPC water molecules by using periodic boundary conditions. The net charge of EP24.15 was compensated by adding three Na^+^ ions. The simulation included 218,640 atoms. The temperature was kept constant at 300 K using the Berendsen thermostat. The particle mesh Ewald method (PME) was used for electrostatic calculations. A non-bonded cutoff of 0.9 nm for Lennard-Jones potential was used. Simulations of covalent hydrogen bonds were delimited by the Shake algorithm. A time step of 2 fs was used. Simulations started with the structure of the human EP24.15, as described in 1S4B, with the added C-terminal 10-residues tail, as described above. Initially, the solvent was relaxed by energy minimization, followed by 15 ps of MD at 300 K, while restraining protein atomic positions with a harmonic potential. Then, the whole system was subjected to minimization by steepest descent and conjugate gradient methods without restraints, and an MD was started by raising the temperature to 300 K by heating the solvent to 300 K over a period of 25 ps. This step was followed by a period of 100 ps for equilibration. Production runs were started at 300 K and simulated for 5 ns. Energy data were stored every 10 time steps, and atomic coordinate values for the entire trajectory recorded were stored every 2 ps.

### Structural Analysis

Solvent accessible surface area (SAS; relative and absolute) was calculated for the selected cysteine residues of human thimet oligopeptidase 24.15 using the structure PDB 1S4B with no water molecules or ions. Relative SAS values are the surface areas of the cysteine residue in the protein relative to that for the exposed residue in the tripeptide Ala-Cys-Ala. In addition, interatomic distances between the SG (sulfur atom) of Cys residues and any positively charged atom up to a 10 Å distance were calculated. All of these calculations were performed using the Sybyl 8.0 (Tripos) program.

### Covalent Docking

The tripeptide glutathione (GSH) was covalently docked onto the 3D structure of human thimet oligopeptidase EP24.15, using the structure PDB 1S4B with no water molecules or ions. This simulation was carried out using Gold, version 5.0, with GA settings similar to those for noncovalent docking and all of the other default docking settings. The results were scored using the Goldscore function. The covalent docking option was chosen, determining that the SG sulfur atom from each of the selected EP24.15 cysteine residues had to be bound to the sulfur atom of glutathione and thus form a disulfide bond between the protein and the ligand. The hydrogen atom bond to the sulfur atom of GSH was removed so that the sulfur atom would not exceed its valence. Ten possible solutions were then obtained. We avoided the automated procedure by turning off the option for the termination of the docking. We had the option of allowing the generation of different structures that were visually evaluated.

## Results

### Identification of EP24.15 S-glutathiolated Cys Residues

**Figure 1:**
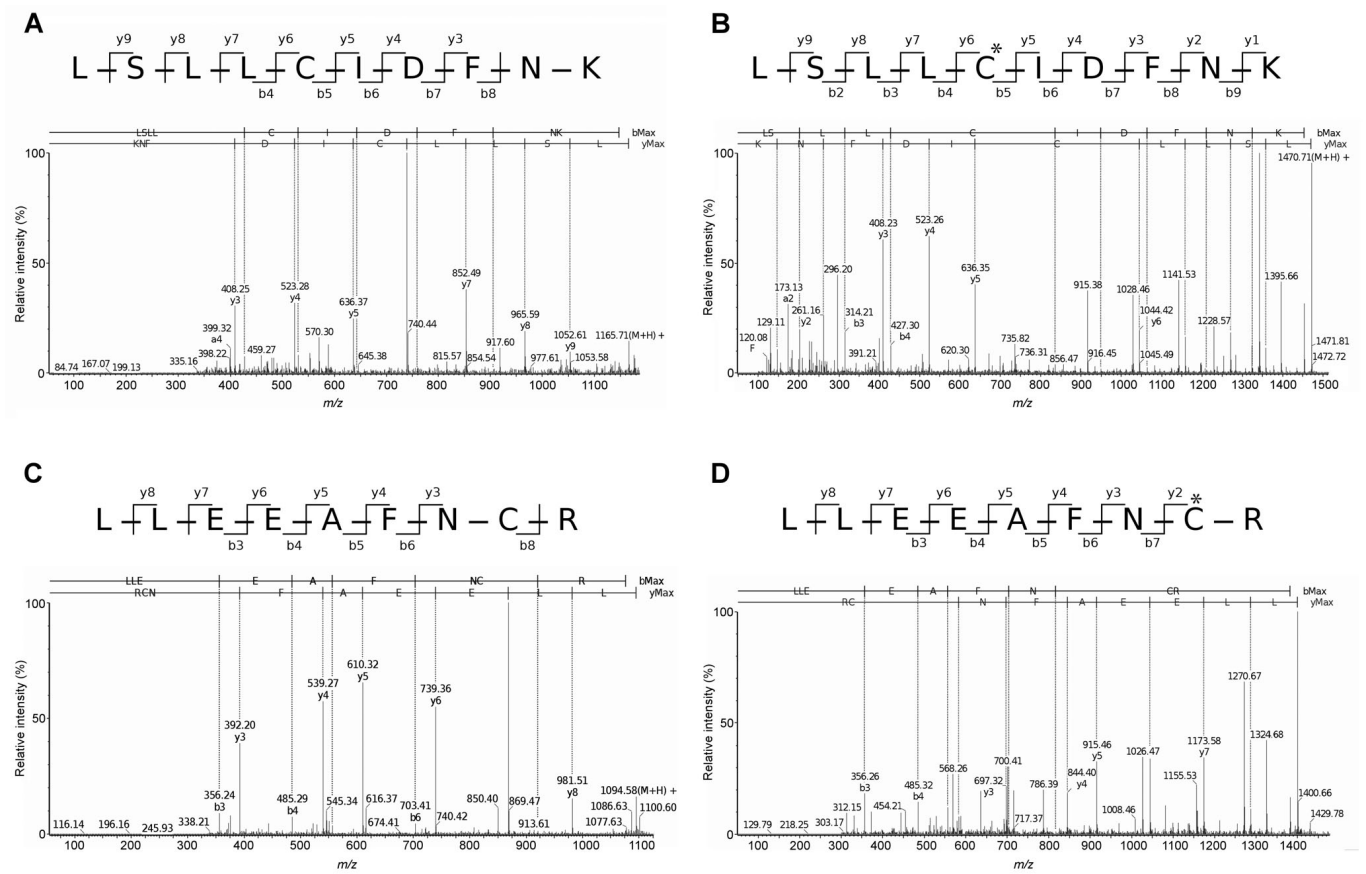
Spectra representative of Q-ToF analyses. The spectra shown were obtained after trypsin-hydrolysis of EP24.15 incubated with TCEP, followed or not by treatment with 1 mM GSSG, and refer to the fragments containing the C175 and C246 residues as follows: (A) and (C) after TCEP-treatment, respectively; (B) and (D) after incubation of the TCEP-reduced protein with 1 mM GSSG, respectively.

The first step was to identify by mass spectrometry (MS) the Cys residues prone to S-thiolation by incubating the TCEP-reduced wild type protein with 50 µM and 1 mM GSSG concentrations. According to the results obtained, five Cys residues were modified (by the addition of 305.1 mass units) after incubation with 1 mM GSSG. The same residues were reduced after TCEP treatment (spectra of C175 and C246 are shown in Fig. 1A and C, respectively). As depicted in Table 1, the S-glutathiolated Cys residues were C46, C175, C246, C682 and C687. Residues C46, C175 and C246 were also S-glutathiolated after incubation with 50 µM GSSG. In addition to the five modified Cys, two fragments containing unmodified Cys residues were found after 1 mM GSSG treatment, residues 427 and 434, as shown in Table 1. The remaining eight Cys were not identified after protein incubation with 1 mM GSSG. The Q-ToF analysis was conducted three times with the 1 mM GSSG-incubated protein. Four of these eight residues (C177; C231; C248 and C253) were detected after TCEP treatment in two out four of the experiments performed. Two other reduced Cys residues (C7 and C18) were detected in one of the experiments, where EP24.15 was treated with 50 µM GSSG. We also searched for other possible sulfhydryl modifications, such as -SOH, SO_2_H and SO_3_H, in all of the fragments containing Cys residues predicted. No other modification was observed, except for the –SH and the –S-SG forms. Figure 1 shows representative spectra of the C175- and C-246-containing fragments generated after EP24.15 incubation with TCEP and 50 µM GSSG.

**Table 1:** EP24.15 fragments containing S-glutathiolated Cys residues generated by trypsin hydrolysis and followed by Q-ToF analysis.

Cys location	Mr(calc)	Delta	Sequence
46^1^	2186.03	−0.02	R.ALTTQLIEQTKCVYDR.V
175^1^	1625.79	0.00	K.RLSLLCIDFNK.N
246^1^	1554.69	−0.00	R.RLLEEAFNCR.C
682 and 687	1811.65	−0.00	K.GLQVEGCEPPAC
427 and 434	1591.74	−0.04	K.YGHAA*C*FGLQPG*C*LR.Q

The wild type protein was treated with TCEP followed by 1 mM GSSG, as described in the Materials and Methods. Next, samples were digested with trypsin for Q-TOF analysis.

Sequence coverage was **63%**. Underlined and *italic* Cys notations were S-glutathiolated and reduced, respectively. Fragments containing the other eight EP24.15 Cys residues were not detected in this condition; those containing oxidized Cys residues to –SOH, SO_2_H or –SO_3_H were also investigated and not detected. Representative Q-ToF spectra detecting 305.1 mass additions are shown in Figure 1.

1 The same residues were also S-glutathiolated after incubation with 50 µM GSSG.

### The Solvent Access Surface and interactions of Cys Residues in the 3D-EP24.15 Structure

The five Cys residues that were S-glutathiolated after incubation with 1 mM GSSG (C46, C175, C246, C682 and C687), the two residues that were reduced (C427 and C434) and the other six Cys residues were analyzed for their Solvent Access Surface (SAS) and for their interactions established with charged residues in the 3D protein structure. The calculated parameters from eight Cys residues are shown in Table 2. According to the parameters analyzed, common to the five S-glutathiolated Cys residues were either the high SAS or the presence of K or R residues close to the Cys-sulfur atom in the 3D structure. The two reduced Cys residues were found to have low or null SAS and no positively charged amino acid in the vicinity of the cysteine (Table 2). Among the other eight Cys residues, only C253 presents high SAS (37.5) when compared to the SAS obtained among the S-glutathiolated Cys. Moreover, a Lys residue was detected, whose charge is a 7.7 Å distance from C253-sulfur atom, the highest found among the S-glutathiolated Cys residues to any positive charge (Table 2). In addition, fragments containing the C253 residue were not detected by the Q-ToF analysis, even in the TCEP-treated protein, and, as discussed next, the GSH covalent docking to C253 was not allowed.

**Table 2:** Cys residues were analyzed according to their Solvent Access Surface (SAS) and distance of the Cys-sulfur atom from positively charged residues.

Cys	Glutathiolation^1^	SAS^2^		Positive residue^3^	Distance^4^ (Å)
		Relative^5^	Absolute (Å^2^)		
046	Yes	0.70	64.0	Arg - 050	5.0
175	Yes	0.25	24.0	Arg - 263	3.5
246	Yes	0.25	22.0	Lys - 249	5.8
427	No	0.04	3.5	No	–
434	No	0.00	0.0	Arg - 436	7.5
682	Yes	0.35	32.5	No	_
687	Yes	1.00	91	Lys-677	7.7
253	No	0.30	37.5	Lys-249	7.7

The methodology of the analyses performed is described in the Materials and Methods.

1 According to MS analyses.

2 Solvent Access Surface.

3 Positively charged residues identified as close to Cys residues shown in the first column.

4 Distance between positive charge and the Cys sulfur atom.

5 The fractional values (Relative) are the surface areas of the residues relative to those of the exposed residue in the tripeptide Ala-X-Ala, where X is the residue of interest.

The data discussed above indicate that high SAS together in the presence of positively charged residues close to the sulfur atom of Cys residues were common and exclusive to the Cys residues that were S-glutathiolated.

### Covalent Glutathione Docking

**Figure 2:**
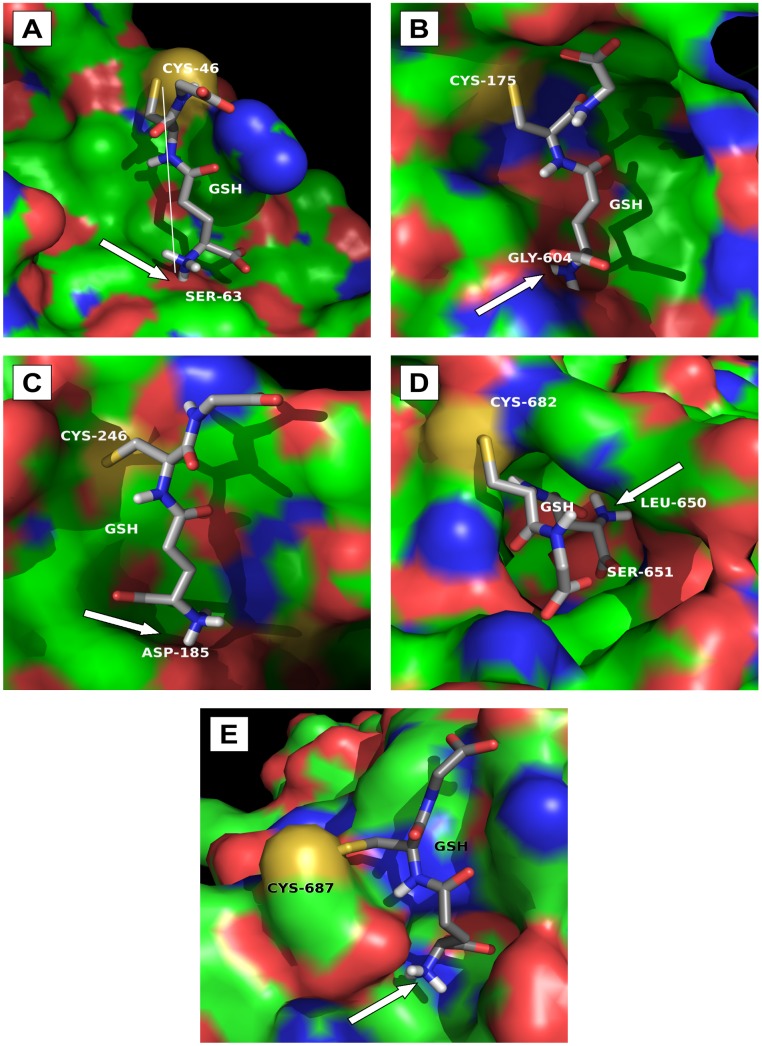
Glutathione covalent docking on EP24.15 surface. The 3D**-**EP24.15 structure focusing S-glutathiolated Cys residues is surface represented and the glutathione (GSH) molecule by sticks. The Cys-sulfur, nitrogen and oxygen atoms are highlighted in yellow, blue and red, respectively. Arrows indicate protein hydrogen-acceptors. Their distance to the protein Cys-sulfur atom is shown in A (white line) and was the same in all other S-glutathiolated Cys residues (B – E).

**Figure 3:**
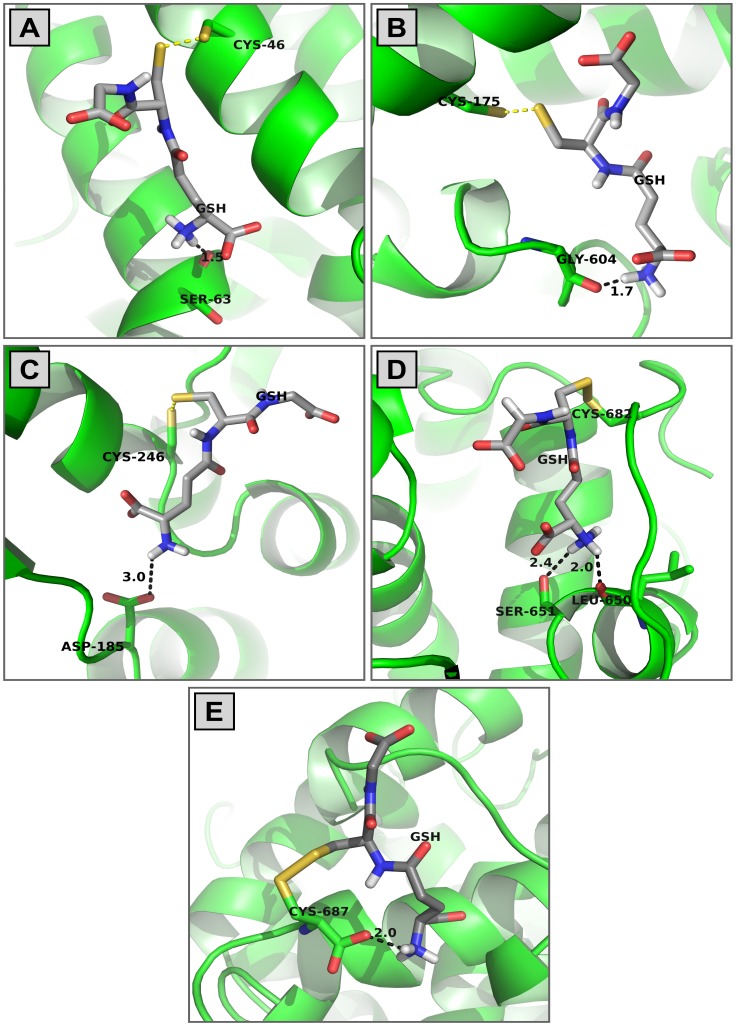
Glutathione interactions to EP24.15 Cys residues. The 3D**-**EP24.15 structure focusing S-glutathiolated Cys residues is represented by ribbons and the glutathione (GSH) molecule by sticks. The Cys-sulfur, nitrogen and oxygen atoms are highlighted in yellow, blue and red, respectively. The distances (Å) of major GSH interactions to EP24.15 residues are highlighted (dashed lines).

In another set of analyses, the covalently attached glutathione (GS) was docked to Cys residues. The five S-glutathiolated Cys residues analyzed (C46, C175, C246, C682 and C687) exhibited pockets in the EP24.15 structure that perfectly accommodated the GSH molecule (Fig. 2A–E). In all of the five cases examined, the glutathione posing was accomplished through polar interactions between the GSH N-terminus and a protein hydrogen acceptor (indicated by arrows), and the distance calculated between the hydrogen acceptor and the Cys-sulfur atom was 11±2 Å (3A-E) in all cases. As illustrated in Fig. 3A, which depicts GSH bonded to Cys46, the GSH N-terminus is 1.5 Å from the backbone oxygen of Ser63. The same polar interaction was observed in the case of the GSH bonded to Cys175 where the GSH N-terminal is 1.7 Å from the backbone oxygen of Gly604 (Fig. 3B). In the cases of GSH bonded to Cys246 and Cys687 (Fig. 3C and E, respectively), the GSH N-terminus interacts (distances 1.8–3.0 Å) with the negative charge on residues Asp185, Glu502 and Cys687 (carboxy terminal). Finally, the N-terminal end of GSH bonded to Cys682 makes two polar contacts of 2.4 and 2.0 Å with the residues Ser651 and Leu650, respectively (Fig. 3D). On the other hand, GSH covalent docking was not allowed in the other nine Cys residues analyzed. The residues C427 and C434 were not S-glutathiolated, because these residues cannot accommodate a GSH molecule in their particular protein bulks and they are not accessible to the solvent (Table 2).

Our major conclusion from the data discussed so far is that the S-glutathiolation of the EP24.15 by GSSG is either dependent on Cys residues’ accessibility to the solvent or on the interaction of the cysteine sulfur atom with a positively charged residue. Moreover, the existence of adequate protein pockets, including hydrogen acceptors for GS-posing, was common to all of the S-glutathiolated Cys residues analyzed.

### Site Specific Mutation

As already known, the Cys thiolate specie is considered much more prone to S-glutathiolation than the protonated thiol because of its higher nucleophilic driving [22], [25]. The formation and stabilization of a given thiolate depends on the thiol pK_a_, which in turn is determined by the protein folding. Depending on Cys residue location into the protein core, the formation and stabilization of the thiolate could be facilitated, in most cases, by a lysine or an arginine residue [25], [26].

Taking the considerations above into account, in the next step, we performed a site-specific mutation of the residue R263 identified as potentially responsible for the maintenance of the thiolate species in the C175 residue selected for the study. Afterward, the ability of EP24.15 mutant protein to undergo S-glutathiolation and oligomerization in the presence of increasing GSSG concentration was analyzed. Finally, by an *in silico* procedure, the interaction between C175 and the mutated residue was analyzed and compared to the wild type protein.

**Figure 4:**
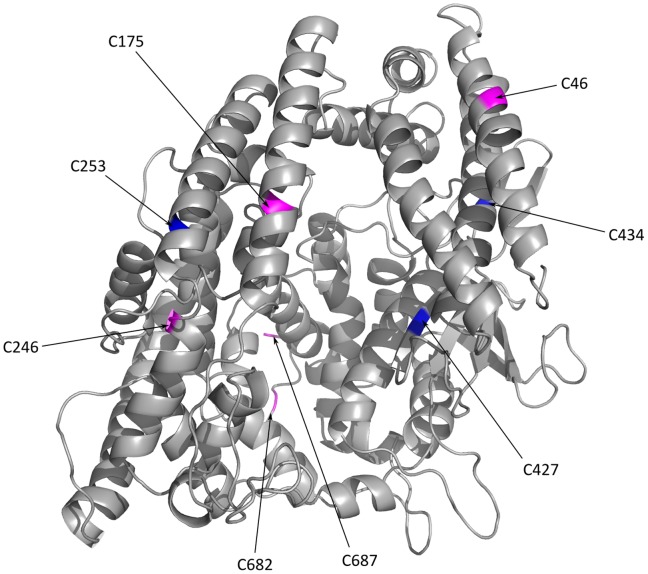
3D-EP24.15 structure. The figure highlights as follows: the five S-glutathiolated Cys residues (46, 175, 246, 682 and, 687; pink); the two reduced Cys residues (427 and 434; blue) identified by MS analyses after incubation of the TCEP-reduced protein with 1 mM GSSG, as shown in Table 1, and the C253 residue (blue) that possesses a high SAS, as shown in Table 2. The catalytic cleft is located on the central structural core where C175 is placed.

The C175 residue is located on the surface of the EP24.15 catalytic cleft (Figure 4) and exposed to the solvent. As observed previously, EP24.15 catalytic activity is modulated by S-glutathiolation [10]. The calculated distance between C175 and the R263 residue was 3.5 Å (Fig. 2S and Table 2), compatible to a saline type interaction [27] favoring the thiolate form of C175. Thus, we chose to mutate the R263 residue to an E residue. Our idea was to completely inhibit any possibility of the formation/stabilization of the C175 thiolate ion.

### GRID Analyses with the R263 Mutant

The calculated distance between the E263 and C175 residues was 6.8 Å, as compared to 3.5 Å for the wild type protein (not shown). Data from the GRID studies on the energies of molecular interaction calculated for the SH probe on the wild type protein and R263E mutant showed that the interaction between the probe and C175 residue was more favorable (−7.8 kcal/mol) for the wild type protein than for the mutated protein (−6.2 kcal/mol). On the other hand, the covalent docking between GSH and C175 that was favored in the wild type protein (Fig. 2B) was also possible in the mutant protein studied (not shown).This result means that neither the Arg residue nor the mutated counterpart are necessary for the interactions established to allow GSH covalent docking to the C175 residue.

### Experimental Data with the R263E Mutant

**Figure 5:**
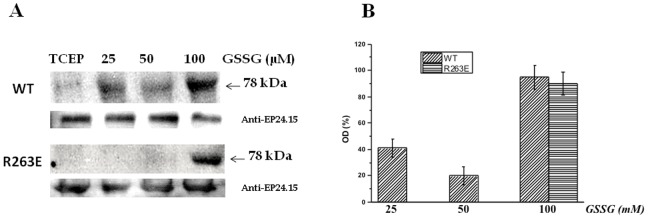
S-glutathiolation of the R263E mutant EP24.15. (A) Blotting shown is representative of anti-GSH labeling of the wild type and the mutant protein after reduction with TCEP, followed by incubation with GSSG at indicated concentrations. The procedure is described in the Materials and Methods. The loading control (anti-EP24.15) was performed by labeling identical membranes utilized for the anti-GSH assay with the anti-EP24.15 antibody. (B) The optical density (OD) of the S-glutathiolated monomeric form of EP24.15 was calculated as the percentage of the OD determined in the anti-EP24.15 blotting, set as 100. Values shown are means ± SD of three independent experiments.

**Figure 6:**
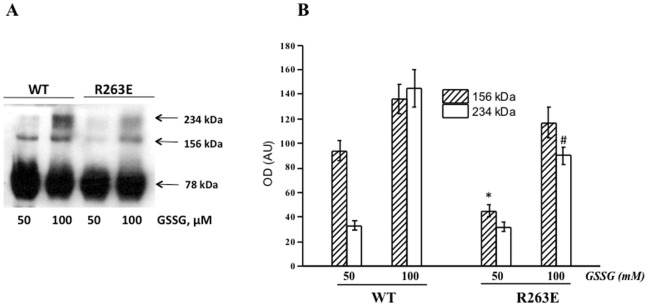
Oligomerization of the R263E mutant EP24.15. (A) Blotting shown is representative of the anti-EP24.15 labeling of the wild and R263E mutant protein incubated at the indicated GSSG concentrations after TCEP reduction. Samples were run on 8.5% SDS-PAGE. (B) Optical density of the dimer and trimer forms of EP24.15. The OD of the 156 kDa and 234 kDa bands was normalized according to the OD values of the 78 kDa band of each sample. *AU*, arbitrary unit. Values shown are means ± SD of three independent experiments. *p≤0.0032 and ^#^p≤0.024 (Student’s t-test) compared to similar WT samples.

Next, experimental assays were performed, showing that EP24.15 S-glutathiolation was greatly reduced in the mutated protein when compared to the wild type protein (Fig. 5). The R263E mutant protein only exhibited S-glutathiolation when incubated at 100 µM GSSG, and this result was reproduced three times. Data obtained on EP24.15 oligomerization revealed that the R263E mutant protein oligomerizes only at GSSG concentrations as high as 100 µM (Figure 6). This result is in agreement with the S-glutathiolation data for the mutant protein (Fig. 5).

The S-glutathiolation of the R263E mutant protein was also analyzed by Q-ToF, after incubation with 1 mM GSSG. Among the five residues that were S-glutathiolated in the wild type protein at same GSSG concentration, only the C246 residue was S-glutathiolated in the mutant protein, though all the fragments containing the remaining four Cys residues were detected.

Altogether, these results indicate that Cys175 likely lost much of its reactivity toward GSSG as a result of the R263 residue mutation, according to the data obtained from the MS analysis. Moreover, the mutated protein does not oligomerize at the same concentrations where the wild type protein oligomerizes, which is in agreement with our proposed mechanism (10). These results suggest that the glutathiolation of a given residue is dependent on the glutathiolation of other residues in the protein. One possibility is that the concomitant S-glutathiolation of the three most reactive Cys residues (those glutathiolated at 50 µM GSSG: C46, 175, and 246) would change the protein structure, modifying the reactivity of the other Cys residues for glutathiolation and oligomerization; however, we do not have data to support this hypothesis. Other mutations should be performed to further test this hypothesis. In the present case, only C246 maintained its ability for glutathiolation in the mutated R263E protein.

## Discussion

Protein S-glutathiolation has emerged as an important post-translational modification. In the first interpretations of the role of this protein modification, S-glutathiolation was considered a means of protecting cellular thiol groups (GSH and protein Cys residues) from oxidation. More recently, it has been discovered that this process is more widespread, capable of modifying protein structure and function, including its role in the cellular anti-oxidative response [11]. Even during homeostasis, cells present a constant pool of protein mixed with disulfides that increases upon an oxidative challenge [28]. The mechanisms of protein S-glutathiolation are relatively well described and demonstrated *in vitro*. There is a consensus on the need for protein thiol reactivity in S-thiolation. The reactivity is dependent on the formation of the thiolate; thus, the process is dependent on the protein thiol pK_a_ (usually within two units of neutral pH). However, thiol reactivity through nucleophilic attack to electrophiles cannot be explained exclusively by the protein thiol pK_a_, as recently discussed in the literature [21]. Either the pK_a_ is important to increase thiolate availability, or the thiolate reactivity toward electrophiles is dependent on the thiol protein bulk [21]. However, the literature indicates a lack of information on the protein structural clues that favor glutathiolation.

The goal in the present work was to analyze the structure and particular features that might favor protein S-glutathiolation through GSSG. We chose the thimet oligopeptidase EP24.15 for this purpose once we had already investigated the mechanism and consequences of its S-glutathiolation [10]. EP24.15 was shown to undergo S-glutathiolation and concomitant oligomerization at physiological GSSG concentrations. However, at GSSG concentrations higher than 100 µM, the oligomerization was inhibited, and the S-glutathiolation was increased. On the basis of these results, we proposed a mechanism for oligomerization through thiol-disulfide exchanges triggered by S-glutathiolation [10].

The present results suggest that at least four prerequisites might be required for EP24.15 glutathiolation to occur through GSSG: (a) the solvent accessibility of the cysteinyl sulfur atom; (b) the stabilization of a thiolate form by positively charged residues close to the Cys; (c) an adequate structural pocket to accommodate the glutathione molecule and (d) interaction of the glutathione molecule with protein residues.

According to the Q-ToF analyses of the fragments generated by trypsin hydrolysis, for the EP24.15 treated with 50 µM and 1 mM GSSG, Cys residues 46, 175 and 246 were glutathiolated through the lowest GSSG concentration, while after protein incubation with the highest GSSG concentration, Cys 682 and 687 also appeared glutathiolated. These findings, as well as the mechanism proposed earlier [10] and previous data reported in the literature [9], strongly indicate that Cys residues 682 and 687 are involved in protein oligomerization. This involvement would explain why, at 1 mM GSSG concentration, the protein does not oligomerize, and why both Cys 682 and 687 were glutathiolated. When incubation was performed with less than 50 µM GSSG, the protein oligomerizes, and C682 and C687 were neither glutathiolated nor in their reduced form. It was previously demonstrated that both Cys 682 and 687 are involved in dimer formation [9], and it is noteworthy that both residues are located in the protein C-terminus and are highly accessible to the solvent (Table 2). Although both conditions favor glutathiolation, most likely the C682 and C687 reactivity toward GSSG might be lower compared to C46, C175 and C246, as these three Cys residues were glutathiolated after incubation with much lower GSSG concentrations (50 µM). The three Cys residues that were glutathiolated at both GSSG concentrations tested (50 µM and 1 mM; residues C46, C175 and C246) did not participate in protein oligomerization unless they had not been glutathiolated after treatment with the lowest GSSG concentration (50 µM). Our hypothesis is that the glutathiolation of these residues confers other functional or structural modifications on the protein, *e.g*., the catalytic activity. Therefore, the Cys 175 is located on the catalytic cleft, and as demonstrated before [10], EP24.15 glutathiolation through a low GSSG concentration (10–50 µM) increases the enzyme activity. Of course, one must predict the involvement of other Cys residues for the oligomerization process as already determined [9]. We cannot exclude the possibility that other Cys are involved in the protein S-glutathiolation, considering that eight Cys-containing fragments were not detected by Q-ToF analyses after incubating the TCEP-reduced wild type protein with 50 µM and 1 mM GSSG and the mutant protein in all of the same conditions.

Amazingly, the R263E mutant presented a decreased capability of oligomerization and glutathiolation after protein incubation with 1 mM GSSG; only the C246 was glutathiolated in the R263E mutant, as cited before. The R263 is the only positively charged residue close enough to C175 to allow the formation and stabilization of a thiolate at C175. Accordingly, the GSSG concentrations necessary for the oligomerization of the mutated protein increased to 100 µM against 10 µM in the wild type protein.

Taken together, the data shown herein and the discussion presented above suggest that although some glutathiolated Cys residues do not participate in the protein oligomerization (most likely residues 46, 175 and 246), their S-glutathiolation appears to be an important trigger of oligomerization. One might suggest that the glutathiolation of those residues confers structural modifications that would facilitate oligomerization through the thiol-disulfide exchange mechanism as proposed [10]. In the case of the C175 residue, it appears that the inhibition of its glutathiolation when the R263 residue was mutated was sufficient to profoundly change the protein structure, as we observed the inhibition of the overall glutathiolation and, as predicted, EP24.15 oligomerization.

Because protein S-glutathiolation is a reversible process and is most likely executed by thioltransferases, especially glutaredoxins [29], this modification is expected to participate in cellular redox modulation, as already proposed in the case of actin polymerization when cells are exposed to growth factors and the proteasome role in the removal of oxidized proteins [19], [30], [31], [32].

## Supporting Information

Figure S1 **Alignment between the primary sequences of the human and rat EP24.15.** Cys residues are highlighted in yellow. Sequences were obtained from NCBI Protein Database and alignment was performed by the BLAST tool.
(TIF)

Figure S2 **EP24.15 partial view.** C175 and R263 residues and the distance between them are highlighted. Data were obtained according to Delano, W. L. (2002) “The Pymol Molecular Graphics System” Delano Scientific, San Carlos, CA, USA. (http://www.pymol.org)
(TIF)
