# Supplementary figures and images for: The Cysteine-Rich Protein Thimet Oligopeptidase as a Model of the Structural Requirements for S-glutathiolation and Oxidative Oligomerization
Source: PLoS One. 2012 Jun 25;7(6):e39408. doi: 10.1371/journal.pone.0039408 (PMC3382611; doi:10.1371/journal.pone.0039408)

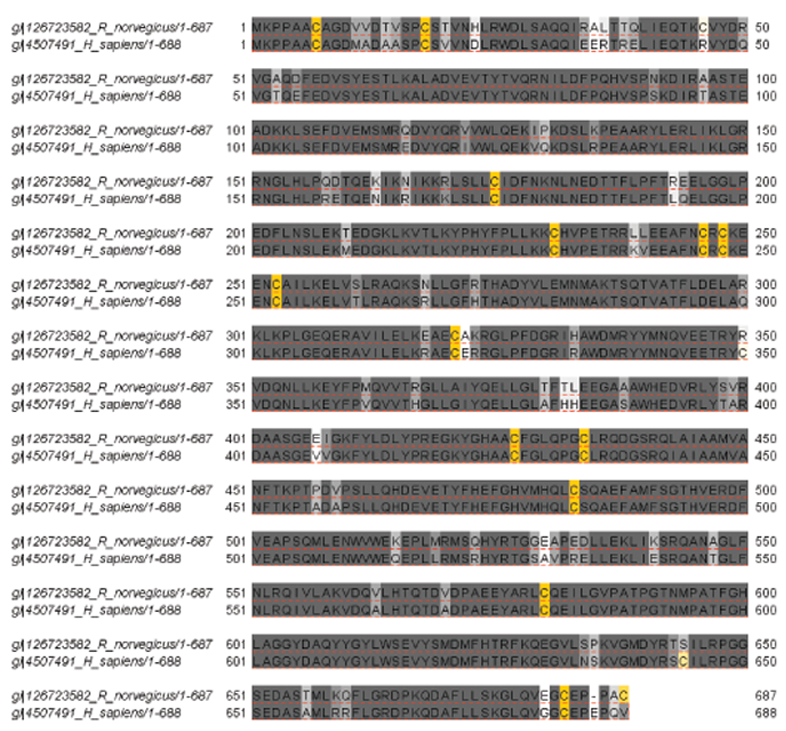

Supplement: Figure S1 — Alignment between the primary sequences of the human and rat EP24.15. Cys residues are highlighted in yellow. Sequences were obtained from NCBI Protein Database and alignment was performed by the BLAST tool. (TIF) [file pone.0039408.s001.tif]

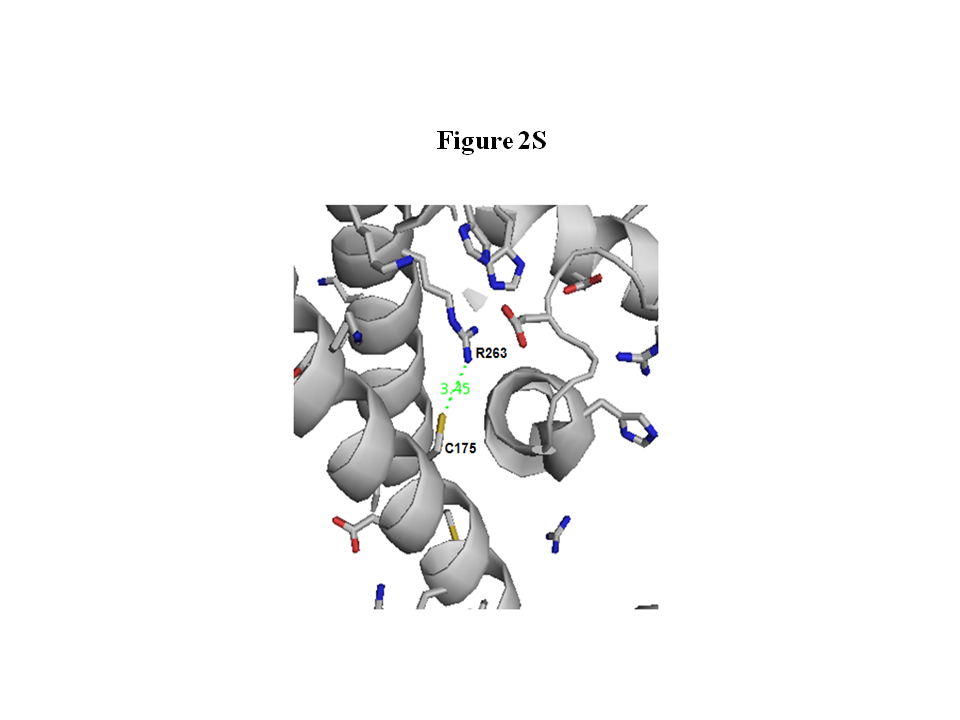

Supplement: Figure S2 — EP24.15 partial view. C175 and R263 residues and the distance between them are highlighted. Data were obtained according to Delano, W. L. (2002) “The Pymol Molecular Graphics System” Delano Scientific, San Carlos, CA, USA. (http://www.pymol.org) (TIF) [file pone.0039408.s002.tif]
